# Selected Quality Parameters of Air-Dried Apples Pretreated by High Pressure, Ultrasounds and Pulsed Electric Field—A Comparison Study

**DOI:** 10.3390/foods10081943

**Published:** 2021-08-20

**Authors:** Artur Wiktor, Aleš Landfeld, Aleksandra Matys, Pavla Novotná, Magdalena Dadan, Eliška Kováříková, Malgorzata Nowacka, Martin Mulenko, Dorota Witrowa-Rajchert, Jan Strohalm, Milan Houška

**Affiliations:** 1 Department of Food Engineering and Process Management, Institute of Food Sciences, Warsaw University of Life Sciences-SGGW, 02-776 Warszawa, Poland; artur_wiktor@sggw.edu.pl (A.W.); aleksandra_matys@sggw.edu.pl (A.M.); magdalena_dadan@sggw.edu.pl (M.D.); dorota_witrowa_rajchert@sggw.edu.pl (D.W.-R.); 2 Food Research Institute Prague, 102 00 Prague, Czech Republic; ales.landfeld@vupp.cz (A.L.); pavla.novotna@vupp.cz (P.N.); eliska.kovarikova@vupp.cz (E.K.); jan.strohalm@vupp.cz (J.S.); milan.houska@vupp.cz (M.H.); 3 Department of Process Engineering, Faculty of Mechanical Engineering, Czech Technical University in Prague, 166 36 Prague, Czech Republic; martin.mulenko@gmail.com

**Keywords:** high hydrostatic pressure, HPP, electroporation, PEF, sonication, US

## Abstract

The aim of this work was to compare selected physicochemical properties of air dried ‘Golden Delicious’ apples, pretreated either by high-pressure processing (HPP), ultrasound (US) or pulsed electric field (PEF). Following parameters of pretreatment were used: HPP–400 MPa for 15 min, US–21 kHz, 180 W for 45 min, PEF–1 kV/cm, 3.5 kJ/kg. The quality of materials was evaluated by their rehydration properties, hygroscopicity, color and total phenolic content. To compare the effectiveness of the utilized methods, determined properties were expressed as relative comparison values against the reference sample obtained without any pretreatment in the same conditions. The performed research demonstrated that properties can be shaped by the application of proper pretreatment methods. For instance, PEF was shown to be the best method for improving water uptake during rehydration, whereas HPP was the most effective in decreasing hygroscopic properties in comparison with untreated dried apples. Among the investigated methods, HPP resulted in the deepest browning and thus total color difference, while the effects of US and PEF were comparable. For all pretreated dried apples, the total phenolic content was lower when compared with reference material, though the smallest drop was found in sonicated samples.

## 1. Introduction

Despite drying being one of the oldest food-preservation and -processing methods, it is still to one of the most popular techniques used in food technology. The global market for dehydrated foods is constantly growing. It is estimated that, in the coming years, it will grow with a CAGR (compound annual growth rate) of 5.3–7.4% [1,2]. Nevertheless, drying as a heat and mass transfer-based process belongs to one of the most energy consuming unit operations applied in food industry, with a share of 12–20% of total energy used in production processes [3,4]. The progress of drying “solid-like” food can be enhanced by the rupture of the cellular structure of the material, and such a technological aim can be achieved by pretreatment of the material. Pretreatment can be performed using mechanical, thermal or nonthermal techniques [5,6,7,8]. High pressure processing (HPP), ultrasounds (US) and pulsed electric field (PEF) treatments are considered to be the most popular and most promising, among nonthermal processing methods, that can be applied to also enhance the mass- and heat-transfer processes [9].

HPP treatment usually involves the treatment of solid and liquid food by pressure, atf 100–800 MPa. Such treatment may result in microorganisms’ and enzymes’ partial or total inactivation, without any (or with negligible) adverse effect on bioactive compounds [10]. Currently, this method is mainly applied at commercial scale, for preservation of juices and smoothies [11]. This is related to the fact that HPP impacts on polymeric structures, stabilized by noncovalent bounds, such as proteins or carbohydrates, but does not have any effect on covalent bounds. High pressure also induces temporary volume changes which, together with changes to the secondary and tertiary structures of proteins, may result in the irreversible loss of cellular structure integrity [12]. The increase of the permeability of cell membranes facilitates mass transfer. Hence, it has been demonstrated previously in literature that HPP can enhance the drying of plants’—such as carrots, apples, green beans [13] or pineapples [14]. The main drawback of HPP as a method, in general, is the high cost of equipment and its batched character. 

US treatment (sonication) is another example of emerging, nonthermal technology. Ultrasounds are mechanical waves, vibrating at frequencies of 0.02–100 MHz. These vibrations can stimulate liquids to inertial and non-inertial cavitation [15,16]. In the case of inertial cavitation, bubbles are formed grow, their volume increases and, at some point, the implode, which generates a shock wave. Usually, for inertial cavitation to happen, the acoustic pressure amplitude must be higher that a particular threshold value. When bubbles do not collapse violently, but instead oscillate in size and volume, the cavitation is non-inertial [17]. Depending on cavitation, the microstructural alterations of tissue material may vary. However, currently it is believed that both types of cavitation may improve membrane permeability [18]. A phenomenon that is closely related to cavitation and which can occur in tissue material is called the “sponge effect”. Mechanical waves that travel throughout material induce oscillating rarefaction and compression of the treated material. Those mechanical changes of the material can be associated with the formation of so-called microchannel promoting of mass transfer within materials exposed to acoustic pressure [19,20]. In the literature, there are many examples of ultrasound’s utilization this way– from extraction and emulsification [21,22], through freezing [23], to osmotic dehydration [16,24,25]. Sonication has been also reported to intensify the drying progress of different tissue materials, such as those of the blackberry [26], raspberry [6], pear [27] or carrot [28]. One of the most important advantages of ultrasound is its relatively low-cost equipment, needed to perform the treatment. However, the main drawback of the sonication of porous material is most probably its low depth of penetration, which makes this method only suitable for thin products [29]. 

PEF treatment involves the utilization of external high-electric-field-intensity short pulses for the treatment of food placed between two electrodes. The exposition of cellular biological systems for PEF leads to reversible or irreversible electroporation. This phenomenon increases the permeability of cell membranes due to the formation and growth of transmembrane pores and/or rupture of cell continuity [30]. There are many examples of successful PEF application in food processing [31]. Ruptured cellular structure results in better extraction of different compounds [32], enhanced freezing [33], osmotic dehydration [34] or preservation of juices [35]. PEF has been also implemented at the industrial scale, mostly for processing potatoes [36]. The literature also provides examples of the positive impacts of PEF pretreatment on drying kinetics and quality of product. For instance, recently it has been demonstrated that PEF reduces the drying time of parsnips [37] and onions [38]. Moreover, the application of PEF can positively influence the quality of dry material, as was exemplified using mango [39]. In comparison with HPP, PEF is a cheaper method, and it can be applied using continuous modes. One of the biggest advantages of PEF is its volumetric character, which makes this technique unique when compared to US. 

HPP, US and PEF allow achieving similar technological aims though different mechanism of action. Thus, the aim of this work was to compare selected physicochemical properties of air-dried ‘Golden Delicious’ apples, pretreated either by HPP, US or PEF. 

## 2. Materials and Methods

### 2.1. Materials

‘Golden Delicious’ apples were used in this research. Only healthy-looking apples with similar dimensions and similar color, without any mechanical injuries, were used. The apples were stored at a temperature of 4–5 °C and. before each experiment, were withdrawn from cold storage and left to equilibrate at room temperature, before being washed with potable water. 

### 2.2. Technological Methods

Samples were pretreated, before drying, using HPP, US or PEF, using the parameters listed in Table 1. The parameters of pretreatment used in the study were selected based upon preliminary experiments (data not shown) related to electrical conductivity and mechanical-properties measurements. Depending on the method, because of technological limitations, samples were either cut into 5 mm thick slices (US, and control) or treated as whole (PEF, HPP) and sliced directly, before drying, into the same-sized cuts. 

#### 2.2.1. High-Pressure Processing (HPP)

**Figure 1:**
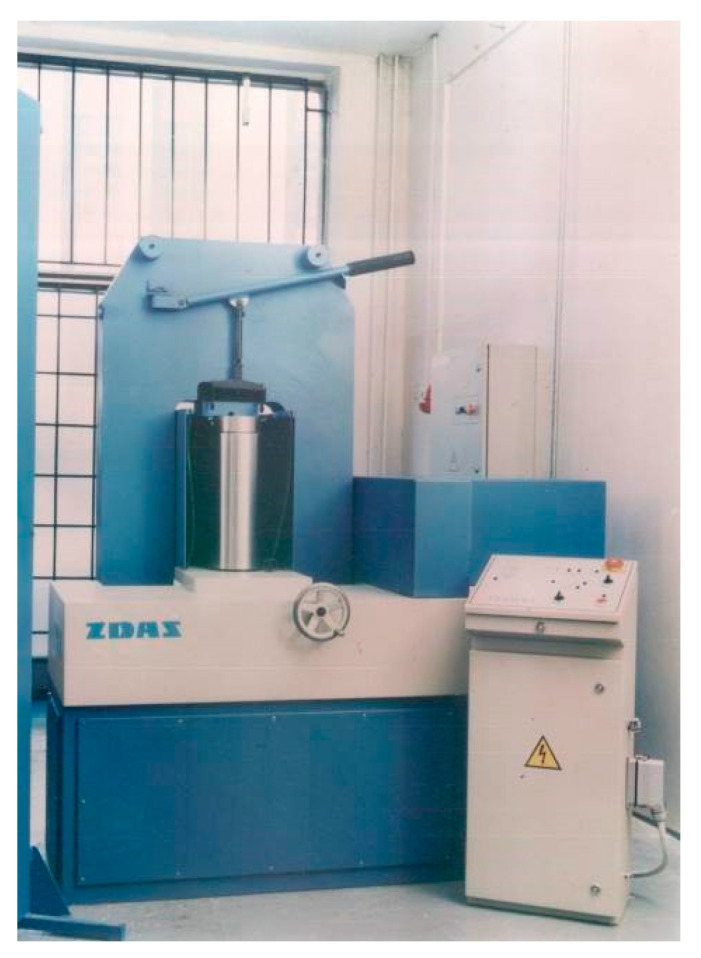
Photography of a high-pressure press CYX 6/0103 (own elaboration).

The treatment of raw whole apples with high pressure was performed in a high-pressure press CYX 6/0103 (ŽĎAS join stock company, Zdar on the Sasau, Czech Republic), which is presented in Figure 1. The volume of the chamber is 2 L, the inner diameter of the chamber is 90 mm, the height is 320 mm and the maximum achievable pressure is 450 MPa. The raw materials were placed in plastic PA/PE bags and filled with potable water (20–25 °C). The bag was then sealed, with a minimum of air inside, and placed in a chamber partially filled with water. Then the upper lid was placed on the chamber and secured with the press frame. The pressure and pressure-holding time were controlled by the system. A pressure of 400 MPa and a holding time of 15 min were chosen for the experiments, based on preliminary research. The temperature of the chamber contents ranged from 21 to 25 °C.

#### 2.2.2. Ultrasounds (US)

Sonication was applied for 45 min, using an ultrasound bath (MKD-3, MKD Ultrasonics, Warsaw, Poland) working at a frequency of 21 kHz and power of 180 W (internal dimensions: 240 × 140 × 110 mm), which corresponded to the ultrasound intensity of 1.8 W per gram of apple tissue. The bath was filled with distilled water of room temperature (20 ± 1 °C). The material was placed in the baker, containing tap water at room temperature (20 ± 1 °C). The ratio between water and material was 1:4. After sonication, apple slices were separated from the water using a sieve and blotted with tissue paper. The parameters of sonication were selected based on preliminary tests.

#### 2.2.3. Pulsed Electric Field (PEF)

A pulsed electric field was generated by the PEF Pilot system (Elea Vertriebs- und Vermarktungsgesellschaft GmbH, Quakenbrück, Germany) at an electric field strength of 1.0 kV/cm and an energy input of 3.5 kJ/kg. The system provided monopolar, near-rectangular pulses with a width of 4 µs. Whole apples (ca. 250 g) were placed in the treatment cell and filled with tap water (21 ± 1 °C) up to 1 kg. Afterwards, the chamber was closed with a special lid to ensure that all apples are covered were completely in water. The gap between the stainless-steel electrodes was 24 cm. After application of PEF, the material was removed from the chamber, dried with tissue paper, and cut into 5 mm-thick slices. The parameters of treatment were selected based on preliminary tests as aforementioned.

The specific energy input *W_p_* was calculated based on the following equation [40]:

*W_p_* = (*U*⋅*I*⋅*t*⋅*n*)/*m* (1)

where *n* is the number of pulses; *m* is the mass of the treated samples (kg); *U* is the voltage (*V*), t is the width of the pulse (*s*) and *I* is the current (A).

#### 2.2.4. Convective Drying

Untreated and pretreated apples were subjected to air drying (convective drying—CD) in a prototype laboratory dryer (Warsaw, Poland or Prague, Czech Republic) at a temperature of 70 °C and air speed of 1.5 ± 1 m/s. The mass of the apples was monitored throughout the process, using a balance coupled to the computer that served as a data logger. Drying was performed until the apples achieved constant mass at least for 15 min, and drying time was expressed as drying needed to reach relative moisture ratio *MR* of 0.02.

Moisture ratios were calculated using following equation [41]:

*MR* = *u*_*τ*_/*u*_0_ (2)

where *u*_0_ is the initial moisture content [kg H_2_O/kg d.m] and *u_τ_* is the moisture content at *τ* moment of the drying [kg H_2_O/kg d.m]. 

### 2.3. Analytical Methods

#### 2.3.1. Dry Matter Content (DM)

Dry matter content was determined using the gravimetrical method according to AOAC procedure [42]. The analyses were done in triplicate. 

#### 2.3.2. Water Activity

Water activity was measured at 25 ± 1 °C, using calibrated instruments (AW Sprint, Novasina; AquaLab, Decagon, Munich, Germany) at least in triplicate.

#### 2.3.3. Rehydration Properties

One slice of dried material was added to a beaker with 100 mL of distilled water at 20 °C for 1 h [43]. After this time, the sample fluid was filtered through a sieve and filter paper. Subsequently, the sample was weighed, and the dry matter was determined. The experiment was repeated at least three times.

According to the equations below, rehydration properties were expressed as dimensionless values of rehydration rate (X) and soluble solids (SSL) losses as a function of rehydration time.

X = *u_rτ_*/*u*_0_ (3)

SSL = (m_τ_ ⋅ dm_τ_)/(m_0_ ⋅ dm_0_) (4)

where m_τ_ is the mass of rehydrated material in time τ of the process [g], m_0_ is the initial sample mass of rehydrated material [g], *u_rτ_* is mass of the water in the sample after time of rehydration τ in [kg H_2_O·kg dm^−1^]; (unit means kg of water per kg of dry mater), m_0_ is mass of water in fresh sample [kg H_2_O·kg dm^−1^] and dm_τ_, dm_0_ are dry matter contents in samples after rehydration time τ, and in dried samples.

#### 2.3.4. Hygroscopic Properties

The hygroscopic properties (H_72h_) of the dried sample were determined by sorption of water vapor by samples placed in an environment with a water-activity value of one for 72 h [43], and expressed as change in material–weight ratios, after 72 h, according to the following equation:

H_72h_ = m_72h_/m_0_ (5)

where: m_72h_ is the mass of material after 72 h of sorption [g], m_0_ is the initial mass of the sample. The experiment was performed at least at three replicates for each analyzed sample.

#### 2.3.5. Color

The optical properties of apples were measured by a reflectance method in CIE L*a*b* scale (CM-5, Konica Minolta, Japan). D65 source of light, 8° angle and a CIE 2° standard observer were set during the measurements [44]. The analysis was repeated at least five times for each analyzed variant. Based on the obtained color coordinates, the total color difference (TCD) was calculated:

TCD = ((ΔL*)^2^ + (Δa*)^2^ + (Δb*)^2^)^0.5^ (6)

where: ΔL*, Δa*, and Δb* are the differences of L*, a*, and b* between untreated or treated dried samples and fresh (raw) apple.

#### 2.3.6. Total Phenolic Content

Total phenolic content was determined according to the methodology described by Nowacka et al. [45], in at least two replicates, for each of the tested variants. In brief, 2 g of dried material was mixed with 80% (*v*/*v*) aqueous ethanol solution and homogenized. Then the homogenized material was boiled, and, after cooling, the extract was filtered to the 50 mL volumetric flask. Then ethanol solution was added to the line obtaining 50 mL of extract. The total phenolic content was measured using 0.18 mL of extract, which was mixed with 4.92 mL of distilled water, 0.3 mL of Folin–Ciocalteau’s reagent, and after 3 min of 0.6 mL of sodium carbonate. The samples were thoroughly mixed between additions of reagents. Samples were stored for 1 h in the dark, and absorbance was measured at 750 nm using spectrophotometer (Helios Gamma, Thermo Fisher Scientific, New York, NY, USA) against sample without addition of extract (blank sample). The obtained results were expressed in mg-of-gallic-acid-equivalents per 100 g of dry matter.

### 2.4. Statistical Methods

Statistical analysis was performed using TIBCO company software (STATISTICA program, version 13, Palo Alto, CA, USA) and Excel (Microsoft, USA) software. Comparison of results between untreated and treated samples was performed using the student’s *t* test. Moreover, cluster Analysis (CA), using Ward’s agglomeration method, and principle component analysis were performed (PCA), taking into account all evaluated relative values. 

## 3. Results and Discussion

### 3.1. Drying Time

**Figure 2:**
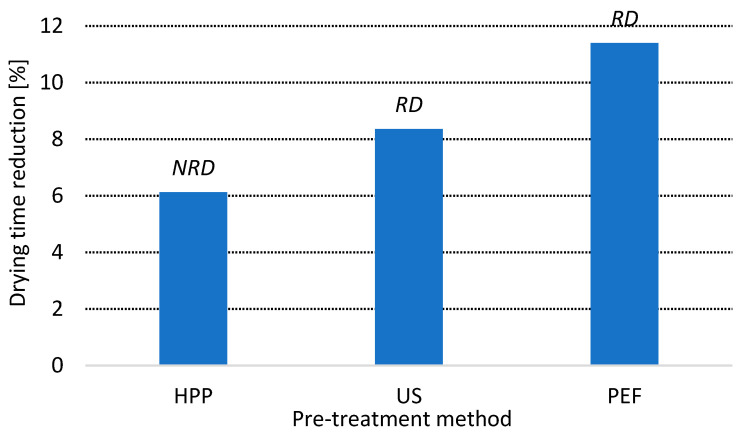
Drying time reduction of HPP, US and PEF treated apples as calculated in comparison with untreated material drying. *RD* indicates statistically significant difference, *NRD* indicates statistically non-significant difference in comparison with untreated dried material (Student’s *t* test, α = 0.05).

Figure 2 presents the reduction of drying time by HPP, US and PEF application. The biggest reduction was found for the process preceded by PEF treatment. In this case drying was shorter by 11.4%, as compared with the reference process. The literature data about the impact of PEF on hot air-drying kinetics is ambiguous. Arevalo et al. [46] did not find any significant impact of PEF on drying time, whereas Wiktor et al. [47] previously demonstrated that electric treatment may decrease convective drying up to 13%. In turn, for other matrices such as carrots the enhancement of drying by PEF reached even 30% in comparison with untreated material [48]. Despite that drying in all cases was carried out at 70 °C the parameters of PEF treatment were different which influent obtained results. Moreover, the physical properties of raw materials subjected for treatment also contributes to the effectiveness of electroporation.

Ultrasounds decreased dehydration time by 8.4% in comparison with control operation. The difference in drying time was significant, from a statistical point of view, for PEF and US alike. More effective process of water removal from apples by ultrasounds application was also stated by Galvão et al. [49] and Nowacka et al. [50]. In both cases, authors explain their results by microchannel formation, which facilitates mass transfer during water evaporation. The reduction of drying time by the application of US was also demonstrated to be effective for matrices other than apples. For example, Abbaspour-Gilandeh et al. [51] showed that US, applied before drying, makes the drying of hawthorn fruits shorter regardless of the drying techniques used. Similar results were also reported by Taghinezhad et al. [52] for kiwi fruits drying. 

The reduction of drying time reached 6.1% when HPP was applied prior to water evaporation. However, the difference was not significant from statistical point of view. In the literature there are some rare examples of HPP’s impact on the drying kinetics of apples. Yucel et al. [13] showed that pressure, applied prior when drying, can decrease the drying time of apples significantly. The size of effect depended on temperature–the higher the temperature, the less visible was the impact of HPP. For instance, when temperature was 85 °C, drying lasted 60.75 and 60.21 min for untreated and HPP (200 MPa, 45 min), respectively. Thus, the results presented in this work are in accordance with data presented previously.

The comparison of the drying times of apples pre-treated by different non-thermal technologies indirectly implies which method and which mechanism of action most probably causes the more severe changes in microstructure. Based on that data, PEF is the most effective in drying enhancement, due to its volumetric character and permeabilization of cell membranes by electroporation process and phenomenon. 

### 3.2. Dry Matter, Water Activity, Rehydration, and Hygroscopic Properties

Raw apple was characterized by high water content. The dry matter was equaled to 13.9 ± 0.1% (data not shown). Drying resulted in a significant increase in the dry substance content for each of the materials and dry matter was in the range of 88.9 to 93.2%. The use of high pressure, ultrasound, and pulsed electric field treatment before drying did not cause any significant changes in the dry matter content compared with the apples that were not pretreated. The results of these tests are presented in Table 2 expressed as relative values of the untreated dried material.

Water activity is a parameter that determines the course of many biochemical processes in food caused by the growth of microorganisms. Water activity below the level of 0.6 prevents the growth of microorganisms and the stability of drought during storage can be maintained [28]. Dried materials were characterized by water activity in the range of 0.228 to 0.249 (data not shown), which was much below the value of 0.6. The samples treated with different pretreatment methods (HHP, US, PEF) significantly differ from untreated dried samples Table 2. In the case of HHP treatment, slightly higher water activity was noticed in the dried material in comparison with untreated dried samples, while for US- and PEF-treated samples, water activity was lower compared with intact dried samples by about 4.42 and 7.23%, respectively. 

Table 2 shows also data related to reconstitution (X-rehydration rate and SSL-soluble solids loss) and hygroscopic properties (H_72h_) dried for apples pretreated with different methods, expressed as relative values calculated regarding the control material (dried, without treatment). Rehydration is a process opposite to the drying process. During rehydration, water enters dried tissues, and the soluble solids of the dry substance move into the water. As a result of rehydration, the weight and volume of the dried materials increase. The changes in tissue structure during the drying process, as well as in pretreatment methods, effects their ability to bind water and inhibit the restoration of the original volume of the raw material [28,53,54]. Its reconstitution properties are essential for the dried material [55]. In the case of the samples subjected to the HHP, US and PEF treatments and convective drying, various changes were observed, depending on the applied pretreatment methods. For HHP treated samples, the dried material was characterized by a significantly lower rehydration rate (X) of about 2.13% and higher soluble solids loss (SSL) of about 9.14%. The results do not confirm previous research by Belmiro et al. [56], which noticed better rehydration properties (up to 2.1 times higher rehydration rate) for dried beans treated with HHP prior drying. 

US application resulted in lower rehydration rates and almost unchanged SSL, however, the results were statistically nonsignificant in comparison with untreated dried material. Usually, in tissue material, ultrasound application affects microstructural changes, and an increase of water uptake during the rehydration process is observed. For example, for carrots subjected to sonication in ethanol solution (up to 3 min), followed by convective drying, the rehydration rate increased up to 19% in comparison with an intact dried sample [28]. Also, for US treatment, prior freeze-drying of button mushrooms, Brussels sprouts and cauliflower resulted in higher rehydration properties [57]. However, with the increasing time of rehydration, a decreasing rate is observed due to the saturation of the material in water [50]. In the case of apple tissue, the treatment was much longer and lasted 45 min. This might negatively influence the microstructure and cause tissue damage, resulting in lowered rehydration ability. 

For the sample subjected to PEF before drying, a significant increase in rehydration rate and decrease of SSL were observed. Additionally, Fauster et al. [58] observed higher rehydration capacity, up to 50%, for PEF pretreated freeze-dried strawberry and red bell-pepper samples. The possible explanation for these better rehydration properties is that electroporation phenomena caused tissue changes and increased the number of pores. Similar effect was also noticed for PEF-treated red bell pepper with prior freeze-drying [59]. As Parniakov et al. [60] stated, PEF application effects shrinkage reduction during the drying process, preserving capillary structures, and affecting rehydration properties.

Hygroscopic materials show the ability to adsorb water in a humid environments, and this causes changes in water content and affects the shelf life of dried materials [41,43]. The structure of the material determines this property, thus in dried apples pretreated with HHP, US and PEF, changes in hygroscopic properties were observed. Generally, porous material shows good hygroscopic properties [28]. The results reveal that only HHP and US significantly changed hygroscopic properties after 72 h of water vapor adsorption. HHP reduced the water vapor adsorption capacity by about 5.51%, which is good, due to the fact that lower water adsorption positively effects the stability of the dried product during storage. In the case of sonicated samples, slightly but significantly increased the water vapor adsorption capacity was observed, while for PEF-treated samples, hygroscopic properties did not significantly differ from untreated dried apples. These results are not in line with those of Rybak et al. [59], who found that US and PEF employed before freeze-drying resulted in an increase of about 42 to 48% in water-vapor adsorption ability. In turn, Zubernik et al. [61] reported that air-dried apple adsorbed 2–3.5-times less water vapor when previously subjected to sonication in ethanol solution (up to 3 min), in comparison with dried untreated tissue. However, in comparison with treatment in ethanol without US, the differences were not significant. Perhaps, the longer US treatment time applied in the current study contributed to significant changes in water-vapor adsorption.

### 3.3. Color

The color of food is generally measured using L*a*b* system, in which L* indicates lightness, a* indicates the color from green (−a*) to red (a*) and b* indicates the color from blue (−b*) to yellow (b*). Based on the alteration of the color descriptors, especially a* and b* parameters, it is possible to predict pigment changes or the occurrence of enzymatic or nonenzymatic browning reactions [62].

**Figure 3:**
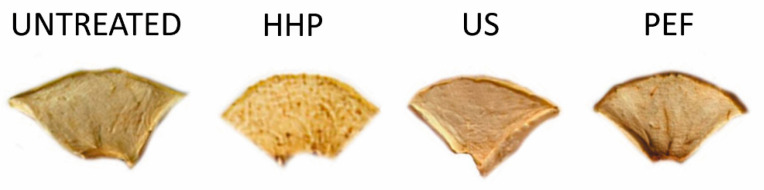
Air-dried apples untreated and treated with HPP, US and PEF.

The relative difference of L*, a*, b* color parameters and total color difference (TCD) between treated and untreated apple tissue are summarized in Table 3 and shown in Figure 3. The changes of the color descriptors were dependent on the type of applied treatment. For instance, the HPP treatment caused significant deterioration of each color parameter, causing darkening of tissue and increase of both a* and b* chromatic parameters. However, the greatest changes were observed in the case of a* parameter, whose value increased after HPP from 3.81 to 10.70, which corresponded to the relative difference of 180.84%. A similar tendency was observed in the case of PEF treatments, for which the highest changes of a* descriptor was noticed, causing a shift in its value from −2.10 to 5.30. The relative difference was than −352.38%. Furthermore, dried PEF-treated apples were characterized by significantly lower L* and significantly higher a*, while the b* value and TCD remained unchanged. Sonication, instead, contributed to significant change of only the a* descriptor. A lower negative value of −2.70 was noted, after US, in comparison with the untreated value (−2.10). Importantly, ultrasound waves did not contribute to the significant darkening of the apples, which probably depended on the disruption of the cells and leakage of their cellular content, which occurred in the HPP and PEF treatments but was less intense for the US treatment. Such results fit previously described results regarding the mechanism of action of ultrasound, which may cause some alterations of cellular structure but without complete degradation of the cell membrane and vacuoles [63]. Wibowo et al. [64] observed significantly higher lightness of the cloudy apple juice after both HPP and PEF treatments in comparison with untreated juice. Moreover, the PEF contributed also to statistically significant increase of a* parameter, whereas HPP increased the b* value significantly. The authors explain that higher L* value caused by PEF was due to partial inactivation of polyphenol oxidase (PPO) and peroxidase (POD) and thermal effect (the outlet temperature was up to 74 °C). However, the enzyme activity after HPP was statistically unchanged, and thus, probably, browning also occurred nonenzymatically after both PEF and HPP. In turn, in the case of apple tissue var. *Ligol* a significant darkening (decrease of L*) of PEF-treated apples, especially at higher electric field intensity (3 and 5 kV/cm), was noted [65]. Some authors suggest that PEF promotes darkening of the tissue due to higher release of PPO and the substrates of enzymatic browning [66]. The higher a* in dried PEF-treated apples, obtained in current study, confirms these assumptions. Fijalkowska et al. [53] reported a significant increase of L*, a* (shift from negative to positive values) and b* values in dried apples, after 30 min of sonication at 21 kHz in, comparison with dried untreated tissue of the *Idared* variety. The browning reactions’ intensities are dependent on the variety of apple and *Golden Delicious* is characterized by a less-intense browning phenomenon [67]. The largest difference in total color difference in comparisons of untreated dried material was found for HPP-treated apples; it was significantly higher, by 103.64%, which limits utilization of this method for drying pretreatment [65].

### 3.4. Total Phenolic Content

**Figure 4:**
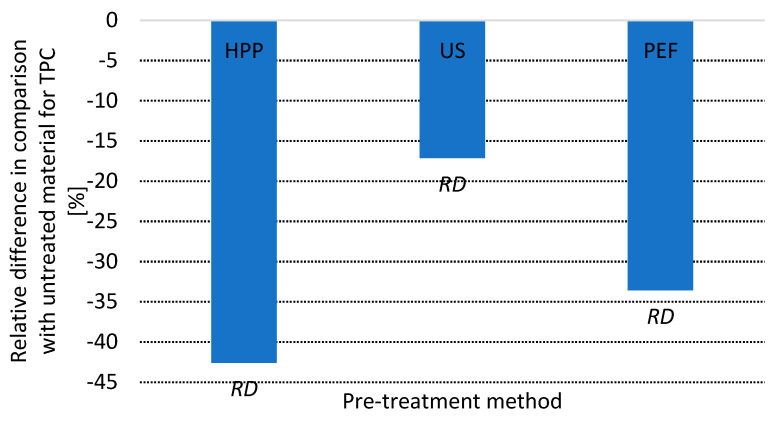
Relative total phenolic content (TPC) in dried apples, pretreated by different methods and calculated in comparison with untreated dried samples. *RD* indicates statistically significant difference, *NRD* indicates statistically non-significant difference in comparison with untreated dried material (Student’s *t* test, α = 0.05).

The application of pretreatment, despite its nonthermal character, resulted in the significant degradation of phenolics in hot air-dried products (Figure 4). The lowest retention of phenolics was found in the case of HPP-treated material, where the loss of total phenolic content, compared with untreated dried apples, was 42.6%. In turn, the lowest degradation of phenolics (17.1%) was found for apples dried with the assistance of US. PEF pretreated dried apples exhibited a total phenolic content lower by 33.6% when compared with the reference material. It is reported that HPP can influence the oxidoreductive enzymes in plant origin material and inhibit residual activity of polyphenol oxidase (PPO) or peroxidase (POD) by 1–33% [68]. However, vast majority of research deals with either juice or enzyme extracts [69], whereas the inhibition of enzymes in solid-like matrices may be different. 

Research performed on peaches showed that PPO activity decreases after HPP, and vacuum packaging from 232 to 106 UE/mg directly after treatment and from 192 to 113 UE/mg after 21 days of storage at 10 °C [70]. Considering that similar situations can apply for apple tissues, the partial inactivation of PPO, rupture of the tissue due to volume changes, degradation of cellular structure and subsequent drying may result in such high degradation of phenolics in dried apples. Similar explanation can be considered as reliable for PEF-treated material, for this method also was reported to cause partial PPO inhibition [70] and, as explained previously, leads to the rupture of microstructures. However, some research indicates that PEF may lead to higher inhibition of PPO and POD than HPP [64] which could partially explain the lower degradation of phenolics found for PEF treated dried apples. Another important factor that can influence such results is the time of treatment. Here, the pressure-holding time, in the case of HPP, was 15 min, whereas the application of PEF lasted less than 1 min. Also, other existing articles in this field confirm the proposed explanation. In fact, there are publications that show that HPP and PEF can lead to a decrease of phenolics in fresh apples [65,71]. Subsequent exposition of such treated materials for conditions that favor oxidation—as it takes place during drying—may only intensify this change. As aforementioned, these results, alongside drying kinetics, indirectly demonstrate that sonication led to the smallest changes in cellular structure, which manifested the lowest degradation of phenolics in the final product. It has to be emphasized that US can also inhibit the activity of oxidoreductive enzymes present in food [72] which, as previously explained for PEF and HPP, can affect the stability of phenolics during drying.

### 3.5. Principle Component Analysis and Cluster Analysis

**Figure 5:**
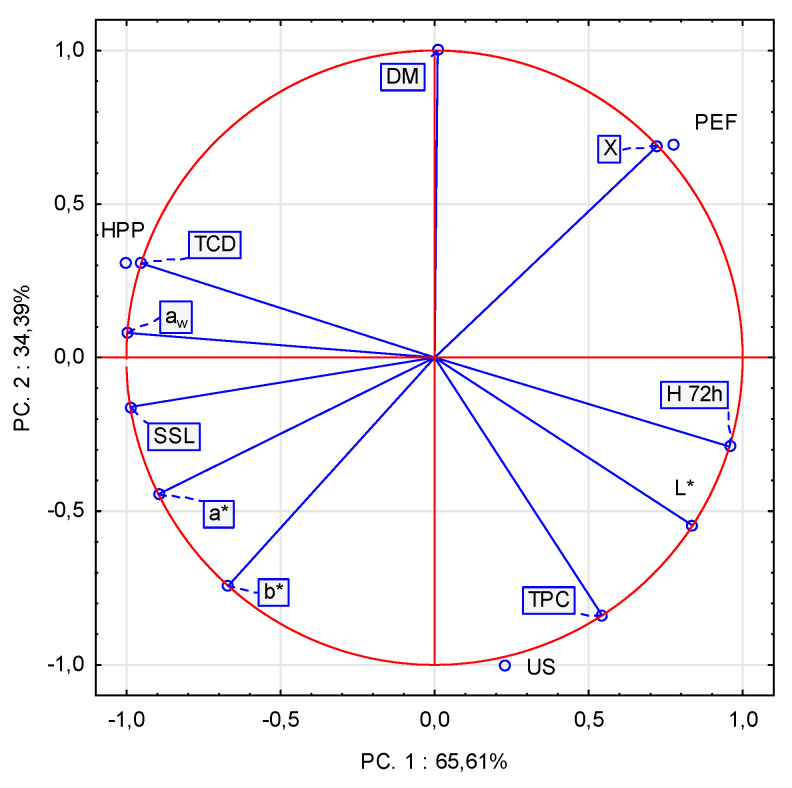
BiPlot of the results of principle component analysis which considered all investigated parameters.

Figure 5 presents the results of PCA in a form of a BiPlot. According to this analysis, the first component (PC1) explained more 65.61% of the variability of the results, while the second (PC2) explained the rest. PC2 was mostly associated with the dry-matter content (DM) and relative water content during rehydration (X), whereas PC1 concerned water activity, soluble solids loss (SSL) and hygroscopicity (H_72h_). Based on the positions of investigated samples and their characteristics it can be stated that PEF material was distinguished from others by its water adsorption capability during rehydration (X), HPP by total color change (TCD) and water activity (a_w_) and US by total phenolics content (TPC). 

**Figure 6:**
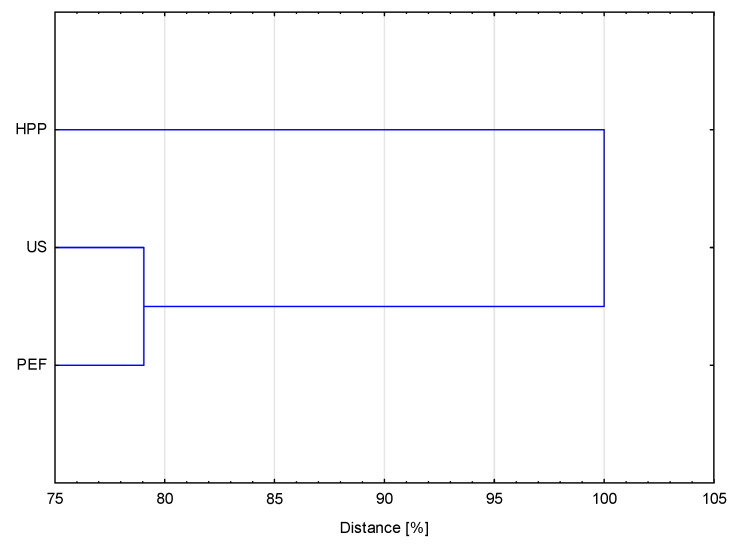
Dendrogram of cluster analysis considering all investigated parameters.

Cluster Analysis (CA) allowed to determine the two investigated groups: one consisted of PEF- and US-treated variants, whereas the second HPP-treated variants (Figure 6). In turn, the distance, which corresponds to dissimilarity, between PEF- and US-treated samples was 79%, meaning that samples subjected to the PEF and US treatments were more like each other than they were like HPP-treated material. Hence, the results of CA correspond to the results of PCA.

## 4. Conclusions

HPP, US and PEF can be used to modify the course of drying and quality of dried products. Among these methods, PEF was the most efficient in reducing drying time or increasing of water adsorption. HPP application prior to drying did not lead to significant reduction of air-drying time, caused the largest color change (browning) of the dried material and the biggest reduction of phenolics, in comparison with untreated material. The high cost of HPP equipment, and its above-listed drawbacks, show that HPP is not necessarily a superior drying-pretreatment method. Nevertheless, the selection of a pretreatment method should be made based on the desired properties of final product and its practical application.

## Figures and Tables

**Table 1:** Basic parameters of pretreatment methods. applied prior to convective drying.

Pretreatment Method	Parameters
HPP	400 MPa, 15 min
US	21 kHz, 180 W, 45 min
PEF	1 kV/cm, 3.5 kJ/kg

**Table 2:** Relative values of water activity, dry matter content, rehydration and hygroscopic properties of dried apples pretreated with different methods, calculated in reference to control (untreated, dried) material.

Pretreatment Method	Relative Difference in Comparison with Control Material [%]				
	X	SSL	H_72h_	a_w_	DM [%]
HPP	−2.13 *RD*	9.14 *RD*	−5.51 *RD*	4.82 *RD*	0.45 *NRD*
US	−2.84 *NRD*	0.4 *NRD*	0.75 *RD*	−4.42 *RD*	−0.54 *NRD*
PEF	28.57 *RD*	−6.76 *RD*	0.84 *NRD*	−7.23 *RD*	0.76 *NRD*

*RD* indicates statistically significant difference, *NRD* indicates statistically non-significant difference in comparison with untreated dried material (Student’s *t* test, α = 0.05).

**Table 3:** Relative values of color parameters of dried apples pretreated with different methods calculated in reference to control (untreated) material.

Pretreatment Method	Relative Difference in Comparison with Untreated Material [%]			
	L*	a*	b*	TCD
HPP	−21.52 *RD*	180.84 *RD*	11.02 *RD*	103.64 *RD*
US	−6.33 *NRD*	28.57 *RD*	12.14 *NRD*	3.08 *NRD*
PEF	−10.55 *RD*	−352.38 *RD*	1.43 *NRD*	3.85 *NRD*

*RD* indicates statistically significant difference, *NRD* indicates statistically non-significant difference in comparison with untreated dried material (Student’s *t* test, α = 0.05).

## Data Availability

The data presented in this study are available on request from the corresponding author.
